# Exacerbated sonic hedgehog signalling promotes a transition from chemical pre-patterning of chicken reticulate scales to mechanical skin folding

**DOI:** 10.1098/rsob.240342

**Published:** 2025-04-16

**Authors:** Rory L. Cooper, Ebrahim Jahanbakhsh, Gabriel N. Santos Durán, Michel C. Milinkovitch

**Affiliations:** ^1^ Genetics and Evolution, University of Geneva, Geneva, Switzerland

**Keywords:** elastic instabilities, self-organization, skin appendages, chicken, reticulate scales, physics of biology

## Abstract

Many examples of self-organized embryonic patterning can be attributed to chemically mediated systems comprising interacting morphogens. However, mechanical patterning also contributes to the emergence of biological forms. For example, various studies have demonstrated that diverse patterns arise from elastic instabilities associated with the constrained growth of soft materials, which generate wrinkles, creases and folds. Here, we show that between days 12 and 13 of *in ovo* development, transient experimentally increased activity of the sonic hedgehog pathway in the chicken embryo, through a single intravenous injection of smoothened agonist (SAG), abolishes the Turing-like chemical patterning of reticulate scales on the ventral footpad and promotes a transition to mechanical labyrinthine skin folding. Using *in situ* hybridization, nanoindentation and labelling of proliferating cells, we confirm that skin surface folding is associated with the loss of signalling placode pre-patterning as well as increased epidermal growth and stiffness. Using additional *in ovo* hydrocortisone treatments, we also demonstrate that experimentally induced hyper-keratinization of the skin mechanically restricts SAG-induced folding. Finally, we verify our experimental findings with mechanical growth simulations built from volumetric light sheet fluorescence microscopy data. Overall, we reveal that pharmacological perturbation of the underlying gene regulatory network can abolish chemical skin appendage patterning and replace it with self-organized mechanical folding.

**Figure:**
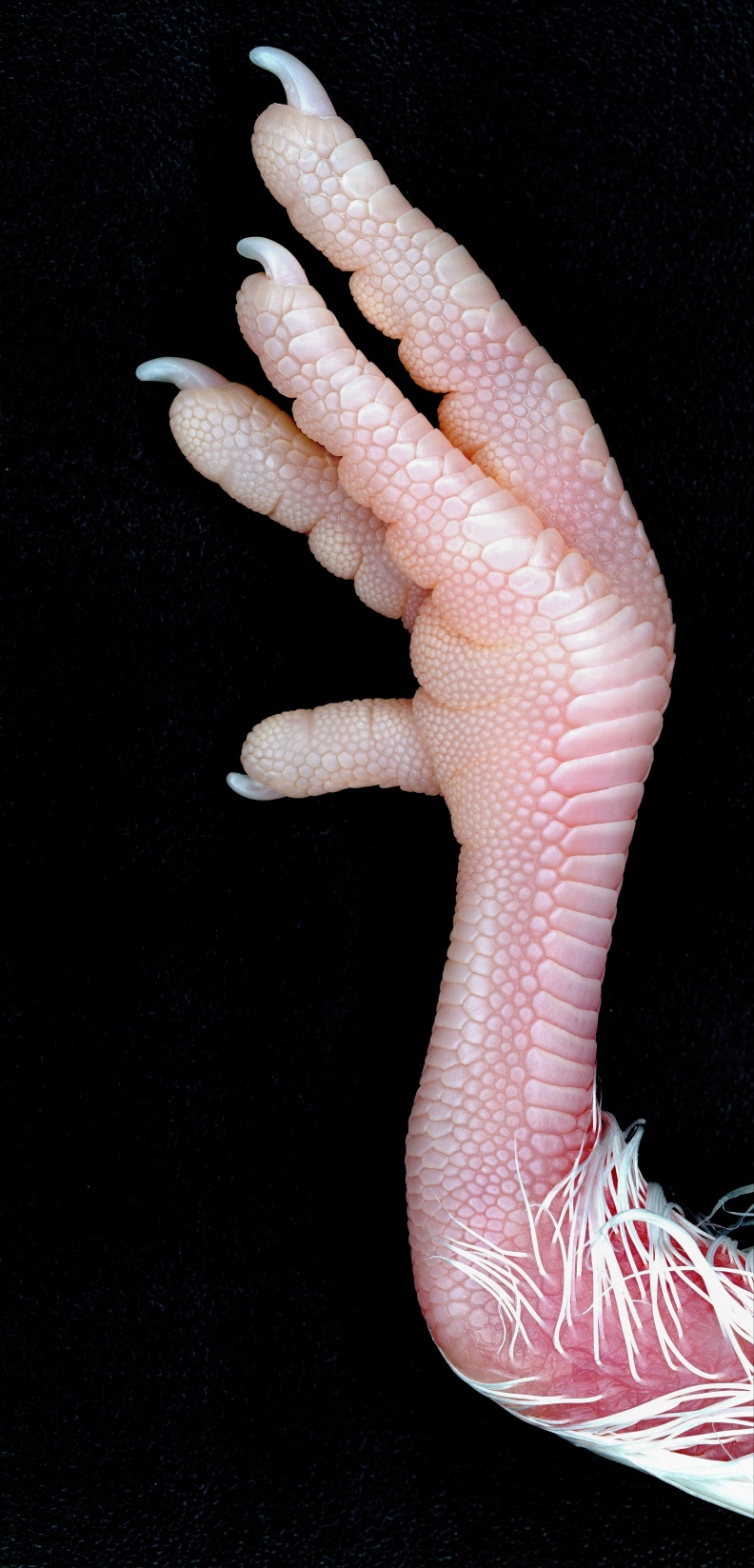

## 1. Introduction

Understanding the mechanistic diversity of embryonic patterning systems remains a key area of biological research. Studies seeking to unravel the chemical (i.e. molecular) systems that mediate such processes have dominated this field in recent years. For example, chemical Turing reaction-diffusion (RD) systems, comprising diffusing and interacting morphogens [1–3], can explain the spatial patterning of skin appendages across diverse taxa, from the scales of sharks and snakes to the feathers of chickens [4–7]. Chemical RD dynamics establish a molecular template defining the punctuated spatial distribution of placodes, which constitute the common foundation of diverse skin appendages and are characterized by conserved local patterns of gene expression [8–11]. Importantly, mechanically-dominated systems, effective at the mesoscopic and macroscopic scales, can also mediate embryonic patterning [12–14], including the development of particular skin appendages [15–18].

Research from the physical and material sciences has demonstrated that intricate and predictable patterns can emerge from the constrained growth or swelling of soft matter [19–21]. In this mechanical process, the differential growth of constituent adherent layers generates elastic instabilities due to the accumulation of a compressive stress field, i.e. the material buckles to produce patterns of wrinkles, creases or folds. These patterns can be both complex and diverse, ranging from stripes and zigzags to labyrinthine segments and even patterns superficially reminiscent of the hexagonal array of feather buds adorning the skin of embryonic chickens [21]. Previous research has revealed that such growth-driven mechanical patterning systems can explain specific aspects of embryonic morphogenesis. For example, work combining both numerical simulations and swellable bilayer polydimethylsiloxane (PDMS) experiments revealed that mechanical instabilities derived from growth-driven compression can recapitulate the emergence of cortical convolutions of the human brain [22]. Furthermore, compressive stress generated from restricted growth of the gut endoderm and mesenchyme underlies the morphogenesis of intestinal villi in the chicken embryo [23,24], and mechanical instability can even drive the morphogenesis of biofilms [25,26]. In such mechanical systems, tissue-layer-specific growth rates and material properties are the key determinants of pattern formation. Overall, mechanical patterning systems are critical features of both embryonic development and the evolution of complex biological shapes and forms [27].

Mechanical patterning processes have also been observed mediating the embryonic patterning of skin appendages. For example, a mechanical system driven by compressive skin folding underpins the irregular polygonal arrangement of crocodile head scales [15,18] and the network of grooves on the naked nose of multiple mammalian species [17]. Furthermore, compressive forces associated with the aggregation of dermal cells also constitute an integral component of the mechanochemical avian feather follicle patterning process [28–31]. In addition, mechanical stresses have been identified as important drivers of both murine hair follicle and tooth development [32,33], as well as in the emergence of the intricate network of crevices that adorns the skin surface of the African elephant [16]. Overall, although chemical systems are key determinants of skin appendage development, integrated mechanical processes are also essential mediators of their patterning across diverse taxa.

We have previously shown that a single *in ovo* intravenous injection [34] of smoothened agonist (SAG) at embryonic day 11 (E11) in the chicken specifically promotes Shh pathway signalling and triggers a complete transition from reticulate scales to feathers on the ventral surfaces of the foot and digits [35]. This transformation is permanent as it does not require sustained treatment and the resulting ectopic down-type feathers subsequently transition into regenerative, bilaterally symmetric contour feathers. Importantly, our RNA-sequencing analyses confirmed that SAG treatment specifically promotes the expression of key Shh pathway-associated genes.

Here, we show that a single intravenous injection of the same drug (SAG) at E12 or E13 (instead of E11) does not trigger a consistent scale-to-feather transition. Instead, this treatment abolishes the chemically mediated Turing-like patterning of reticulate scales and replaces it with mechanical folding of the skin driven by increased epidermal growth and differentiation [36,37]. This results in the emergence of intricate folding patterns on the ventral surface of the footpad. Using *in situ* hybridization (ISH), we show that this folding pattern is not caused by a labyrinthine distribution of local placode-associated gene expression. We next use nanoindentation and reveal that the emergence of folding is accompanied by increased epidermal stiffness. Furthermore, we demonstrate that *in ovo* hydrocortisone treatment induces hyper-keratinization of the epidermis, thereby substantially perturbing the development of reticulate scales. When E12 or E13 chicken embryos are subjected to a combined treatment of SAG and hydrocortisone, SAG-induced folding becomes largely restricted by this hyper-keratinization of the epidermis. Next, we use light sheet fluorescence microscopy (LSFM) to precisely quantify the three-dimensional (3D) geometry and growth of the different tissue layers that comprise this mechanical patterning system. Using this data, we build a mechanical simulation of differential-growth-driven patterning, thereby validating our experimental results. Finally, we use swellable PDMS bilayer experiments to simulate the emergence of patterning observed in our *in ovo* and numerical experiments. Overall, we demonstrate that pharmacological *in ovo* exacerbation of Shh signalling in E12 or E13 chicken embryos abolish chemically mediated reticulate foot scale patterning and replace it with a mechanical patterning process.

## 2. Results

### 2.1. Normal development of avian skin appendages

**Figure 1.**
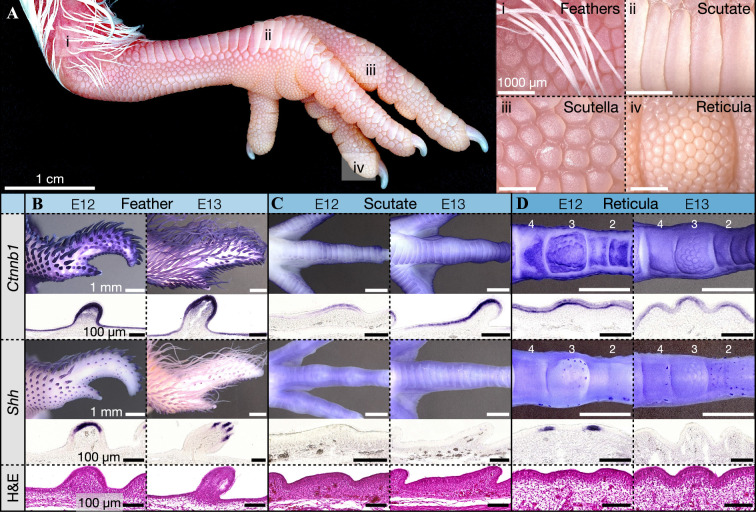
Normal development of avian skin appendages. (A) Skin appendages of the chicken include feathers (i), overlapping scutate scales (ii), scutella (iii) and reticulate scales (iv). Despite their morphological diversity, these appendages share similar early-developmental molecular signalling. (B*–*D) We used WMISH and H&E staining to investigate their development. (B) By E12, *Ctnnb1* and *Shh* expression marks feather buds covering the wing. These buds become long and filamentous by E13, with cryosections revealing diffuse expression of *Ctnnb1*, and *Shh* expression in longitudinal stripes associated with feather branching morphogenesis [38,43]. H&E stained cryosections show feather bud outgrowth from E12 to E13. (C) By E12, scutate scales undergo morphogenesis and express diffuse *Ctnnb1*, although *Shh* expression is absent. H&E staining also shows the progression of their asymmetric growth from E12 to E13. (D) Reticulate scale signalling placodes first emerge at E11 [35] and become evident at E12 on the first, third and fifth toepads, stained with diffuse *Ctnnb1* and highly localized *Shh* expression (sections in the third toepad are shown). By E13, signalling placodes extend to the second and fourth toepads. H&E staining reveals the outgrowth of reticulate scales into their radially symmetric form. Labels ‘2–4’ in (D) refer to the toepad number.

The molecular development of avian skin appendages has been well characterized, with feathers, in particular, providing an important model for understanding the molecular and mechanochemical systems governing their embryonic patterning and morphogenesis [4,30,35,38–40]. In addition to feathers, avian skin appendages also feature various scale types (figure 1A) [35,41]. These include large, overlapping ‘scutate scales’ which adorn the anterior metatarsal shank and dorsal foot surface (figure 1Aii), ‘scutella’ found on the lateral surface of the shank and digits (figure 1Aiii), and radially symmetric nodular ‘reticula’ (also called ‘reticulate scales’) on the ventral footpad and digits (figure 1Aiv) [35,42]. Using a combination of ISH and haematoxylin and eosin (H&E) staining, we examine the normal development of these skin appendages in the chicken at embryonic days 12 and 13 (E12 and E13; figure 1B–D).

Distinct avian skin appendage types exhibit conserved molecular signalling during their patterning and morphogenesis, with the local expression of both β-catenin (*Ctnnb1* gene) and sonic hedgehog (*Shh*) marking placodes [35,42]. Feather placodes first emerge at E7 in a bifurcating dorsolateral row [4]. At E12, feather buds expressing *Ctnnb1* and *Shh* cover the wing surface (figure 1B). By E13, these units become increasingly elongated and filamentous, with *Shh* highlighting longitudinal expression domains associated with the morphogenesis of feather barb ridges [38,43] (figure 1B). H&E staining reveals the progression of feather bud outgrowth from E12 to E13 (figure 1B, bottom panels). Second, scutate scale placodes appear at E10 [35,42] and exhibit diffuse *Ctnnb1* expression throughout their morphogenesis from E12 to E13, although *Shh* expression is absent at these late stages (figure 1C). H&E staining reveals the polarized growth associated with their overlapping, rectangular morphology (figure 1C, bottom panels). Third, reticulate scale signalling placodes are first observed at E11 [35,42]. By E12, these placodes are visible on the third toepad, exhibiting diffuse expression of *Ctnnb1*, and concentrated, yet transient, expression of *Shh* (figure 1D). By E13, additional placodes propagate across the second and fourth toepads. H&E staining reveals the progression of reticulate scale outgrowth, into their nodular, radially symmetric form (figure 1D, bottom panels). Note that, although reticulate scales are associated with placode-like signalling, the presence of an anatomical placode (with its characteristic epidermal thickening due to columnar cells and its dermal condensate) has not been clearly established in reticulate scales. Overall, despite their diversity in form, avian skin appendages exhibit conserved gene expression patterns associated with placode emergence and early morphogenesis [10].

### 2.2. *In ovo* SAG treatment at E12 or E13 results in labyrinthine patterns on the ventral footpad

Having examined the normal development of avian skin appendages, we next aimed to experimentally manipulate this embryonic system, specifically by agonizing the Shh pathway. Indeed, the SHH ligand acts as a conserved morphogen in various developmental processes, including both limb bud and feather patterning [35,38,40,44], and is known to trigger epidermal growth and differentiation [45]. Canonical Shh pathway signalling begins with the SHH ligand binding to its receptor, Patched1 (PTCH1), reversing the repression of smoothened (SMO) and triggering subsequent downstream transcription [36,37]. Upregulation of Shh pathway signalling [35] can be achieved through the use of the SMO agonist (SAG) [37]. We have previously shown that a single *in ovo* intravenous injection of SAG in chicken embryos at E11 triggers a permanent reticula-to-feather transition. We reason that a similar treatment at later stages is likely to increase overall epidermal growth [45] and trigger the accumulation of a compressive stress field in the skin, thereby generating mechanical instabilities [19–21].

**Figure 2.**
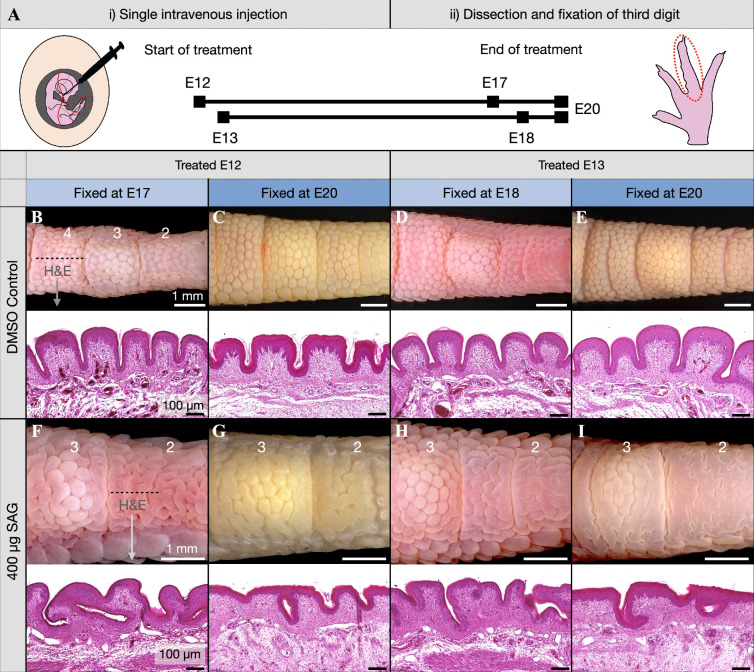
*In vivo* SAG treatment induces the development of labyrinthine patterns on toepads. (A) Embryos were treated at E12 or E13 with a single intravenous injection of either DMSO (as a control) or SAG (300 µg or 400 µg). (B–E) Control samples exhibit normal development of reticulate scales (B,C) when injected with DMSO at E12 and fixed at E17 or E20, and (D–E) when injected at E13 and fixed at E18 or E20. H&E sections of the fourth toepad of control samples illustrate the normal morphology of reticula. (F–I) All samples exhibit extensive labyrinthine surface patterns on the second and fourth toepads (F,G) when injected with SAG at E12 and fixed at E17 or E20, and (H,I) and when injected at E13 and fixed at E18 or E20. H&E sections of the fourth toepad of SAG-treated samples reveal dramatic labyrinthine tissue folding. Labels ‘2–4’ refer to the toepad number. The dashed line in (B) and (F) indicates the location of the H&E sections.

Chicken embryos were treated (figure 2A, table 1) with a single intravenous injection of SAG (or dimethyl sulfoxide, DMSO, as a control) during reticulate scale development at either E12 or E13. In accordance with our previous study [35], we adjusted the SAG dosage according to embryo weight, with E12 embryos receiving 300 μg and E13 embryos receiving 400 μg, equating to 40 μg SAG per gram of approximate embryo weight. Embryos were fixed either 6 days after treatment (i.e*.* at E17 or E18, respectively) or at E20, which is the day before hatching. DMSO control samples exhibit normal development of nodular reticulate scales when injected at E12 and fixed at E17 or E20 (figure 2B,C), as well as when injected at E13 and fixed at E18 or E20 (figure 2D–E). On the other hand, when treated with SAG at either E12 or E13, a labyrinthine pattern emerges on both the second and fourth toepads (figure 2F–I; electronic supplementary material, figure S1). Conversely, the first, third and fifth footpads exhibit mostly unaffected reticula because their development is relatively more advanced at E12 (figure 1D; electronic supplementary material, figure S1)—i.e. at the time of SAG treatment—preventing the shift from the chemically mediated to the mechanical patterning process. Note that the labyrinthine pattern is maintained at E20, at which point the skin tissue appears notably more keratinized (figure 2G,I). SAG treatment causes the skin of the second and fourth toepads to exhibit a dramatically convoluted pattern as shown by the apparent topological changes on two-dimensional (2D) histological sections (figure 2F–I). Note that individual variations in embryonic staging at the time of treatment explain the infrequent observation of similarly perturbed skin patterning on the lateral digit surface in the scutella-forming region of samples treated at E12 and fixed at E17 (electronic supplementary material, figure S2).

**Table 1.** Summary of replicates of SAG injections (figure 2; electronic supplementary material, figure S1).

treatment	DMSO survival (no.)	SAG survival (no.)	treatment effect (%)
E12–E17	8/9	12/18	100
E12–E20	3/3	9/15	100
E13–E18	8/8	12/17	100
E13–E20	3/3	9/14	100

Overall, these results demonstrate that *in ovo* SAG treatment in the embryonic chicken at either E12 or E13 aborts the Turing-like chemical patterning of reticulate scales and triggers the formation of a labyrinthine folding pattern reminiscent of those that emerge from constrained growth-driven mechanical instability [19–21]. However, such a modified pattern of the skin could also be explained by localized skin growth superposed to a modified (labyrinthine) template of gene expression.

### 2.3. The emergence of labyrinthine patterning is not associated with redistributed gene expression

We next sought to determine whether the SAG-induced labyrinthine pattern of the toepad skin is produced by compressive folding through homogeneously increased skin proliferation, or by local skin growth that matches a corresponding labyrinthine pre-pattern of placode markers. Avian skin appendage patterning emerges in accordance with chemical RD dynamics [4,35] integrated with mechanical cues [28–30], and alterations to the morphogens that mediate such systems can result in dramatic shifts in patterning. For example, it has previously been shown that the potent Shh inhibitor cyclopamine [37] can disrupt feather follicle patterning *ex vivo*, resulting in a labyrinthine pattern of *Shh* expression [46]. Similarly, we have recently shown that an intravenous injection of sonidegib, a potent and specific Shh pathway inhibitor, at E9 temporarily produces striped domains (instead of spots) of Shh expression in the skin [43]. In principle, such a redistributed template of gene expression could produce the labyrinthine skin pattern that we observe in SAG-treated embryos (figure 2F–I). Therefore, we undertook whole mount ISH of a series of embryonic tissue fixed at daily intervals following treatment at E13 with either SAG or DMSO as a control.

**Figure 3.**
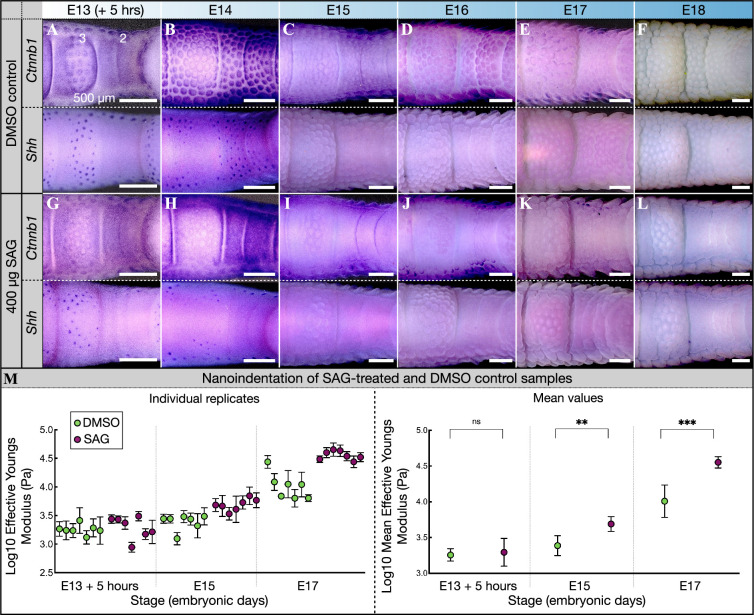
The emergence of a labyrinthine pattern does not correlate with a corresponding local gene expression pattern and is accompanied by increased epidermal stiffness. See also electronic supplementary material, figures S3 and S4. (A–F) Samples injected with DMSO at E13 were fixed every 24 h following injection. (A) At E13 (+5 h), *Ctnnb1* and *Shh* expression stains placodes mostly on the first and third toepads in DMSO control samples. (B) These placodes extend across the second and fourth toepads at E14. (C–F) Reticula outgrowth progresses from E15 until E18, at which point they exhibit their final nodular radially symmetric form. (G*–*L) Samples injected with SAG at E13 were fixed every 24 h following injection. (G) At E13 (+5 h), expression of *Ctnnb1* and *Shh* is similar to that observed in controls. (H) However, by E14 the propagation of signalling placodes is restricted (they do not emerge on the second and fourth toepads), although some peripheral units may appear. The second and fourth toepads remain free from signalling placodes at E15 and E16 (I–J), and the skin surface labyrinthine pattern is visible by E17 and E18 (K–L). We do not observe a labyrinthine local gene expression patterns of *Ctnnb1* or *Shh*. (M) Nanoindentation shows that tissue stiffness of the fourth toepad increases at a significantly faster rate in SAG-treated samples than in DMSO controls. Mean values (± SD) for individual embryos (left panel) and the mean (± SD) of means are shown (right). Labels ‘2−4’ in (A) refer to toepad number.

Embryos injected with DMSO exhibit normal reticulate scale development, with *Ctnnb1* and *Shh* expression highlighting placodes covering the third toepad at E13 (5 h post injection) and propagating over the second and fourth toepads at E14 (figure 3A–B; electronic supplementary material, figures S3–S4). By E15, the morphogenesis of reticulate scales in control samples is advanced, with these units becoming elevated across all toepads and exhibiting their final nodular form by E17 (figure 3C–F; electronic supplementary material, figures S3–S4). The reticulate scale development of SAG-injected embryos is comparable with DMSO controls at E13 (+5 h) (figure 3G). However, SAG treatment inhibits subsequent placode development as they fail to propagate across the second or fourth toepads at E14 or later (figure 3H–I; electronic supplementary material, figures S3–S4). Reticulate scales are mostly absent from these toepads, although some peripheral units remain (figure 3I–J; electronic supplementary material, figures S3–S4). From E17 to E18, labyrinthine patterns emerge on the second and fourth toepads without the development of a corresponding spatial distribution of *Ctnnb1* or *Shh* (figure 3K,L; electronic supplementary material, figures S3–S4). Hence, the observed labyrinthine folding pattern is not associated with a corresponding labyrinthine local expression of classical placode markers, and no fusion of reticulate scales is observed at any time point following SAG treatment.

### 2.4. SAG treatment increases stiffness of the toepad skin surface

If SAG treatment at E12 or E13 only abolished the self-organized Turing-like steady-state pattern of placode-determining morphogens (such as *Shh*), one would expect to observe a smooth skin surface on the toepads of treated embryos. However, the presence of a labyrinthine pattern (figure 2F–I) additionally requires exacerbated overall growth and differentiation of the skin, which is a likely effect of promoting *Shh* signalling [45]. We tested such an effect using nanoindentation [47] to compare changes in toepad skin surface stiffness of SAG-treated versus control chicken embryos (figure 3M).

Measurements of effective Young’s modulus (pascals, Pa) were taken from the fourth toepad of both SAG-treated and DMSO control embryos (see §4 for further details). Both the mean (and standard deviation) of stiffness across multiple measurements within individual embryos (figure 3M, left panel) and the overall mean values (with mean standard deviation) across different embryos of the same stage (figure 3M, right panel) are shown. The data have been log-transformed to normalize the distribution prior to statistical analysis (see §4 for further details). We observe a significant increase in tissue stiffness across embryonic stages (ANOVA with stage and treatment as fixed effects; *F* = 168.28, df = 2, *p* < 0.001) but also due to SAG treatment (*F* = 38.73, df = 1, *p* < 0.001). Indeed, although there is no significant difference in stiffness between the SAG and DMSO control embryos at E13 (+5 h) (mean difference of 0.022 in log Young’s modulus, adjusted *p*‐value = 1.000), SAG-treated embryos exhibit significantly higher stiffness values at E15 and E17 (mean difference of 0.31, adjusted *p*‐value = 0.005; and 0.53, adjusted *p*‐value<0.001, respectively). In addition, we performed labelling of proliferating cells with 5-ethynyl-2′-deoxyuridine (EdU) and observe significantly higher proliferation of the epidermis (electronic supplementary material, figure S5A), but not of the dermis (electronic supplementary material, figure S5B) in SAG-treated samples relative to controls. Overall, our results from nanoindentation and EdU labelling reveal that SAG-treated embryos exhibit greater skin surface tissue stiffness and greater epidermal growth than DMSO control samples.

### 2.5. Hydrocortisone-induced epidermal hyper-keratinization restricts mechanical skin folding

**Figure 4.**
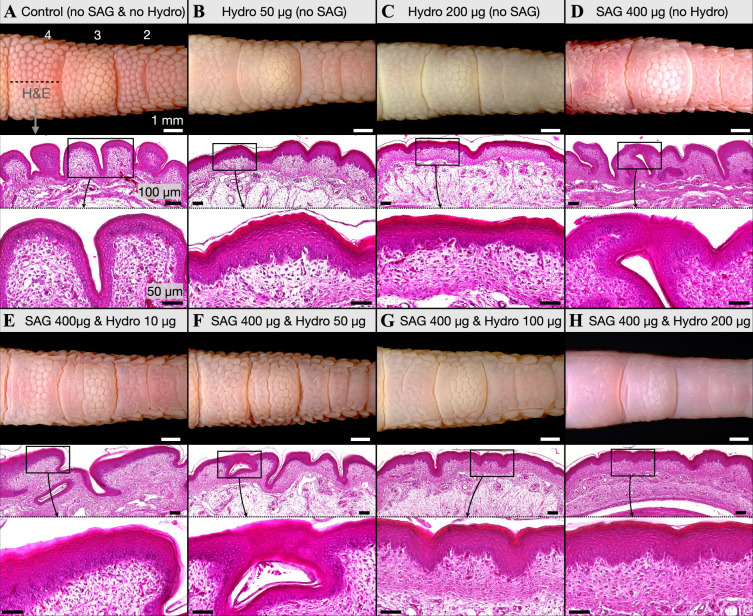
Hydrocortisone treatment constrains SAG-induced folding via increased skin surface keratinization**.** (A) DMSO-injected control embryos exhibit normal reticulate scale development. (B) When treated with 50 µg hydrocortisone, reticulate scale elevation is restricted, and a substantial increase of skin surface keratinization is observed in H&E sections of the fourth toepad. (C) Reticulate scale development is further restricted when embryos are injected with 200 µg hydrocortisone, with toepads appearing flat and highly keratinized. (D) When injected with 400 µg SAG, extensive folding emerges (see also figure 2F–I). (E,F) Some skin folding is also observed in embryos jointly treated with 400 µg SAG and either 10 µg or 50 µg hydrocortisone. (G) However, folding in embryos treated with 100 µg hydrocortisone and 400 µg SAG is restricted and we note a dramatic increase in skin surface keratinization. (H) Folding is virtually inexistent in samples treated with 200 µg hydrocortisone and 400 µg SAG, with toepads appearing mostly smooth and highly keratinized. Labels ‘2–4’ in (A) refer to the toepad number.

All results presented above strongly suggest that the labyrinthine skin surface pattern, observed on the ventral toepads of chicken embryos treated with SAG at E12 or E13 (figure 2F–I), corresponds to the emergence of elastic instabilities (i.e. mechanical folding). This is due to the exacerbated growth and differentiation of the epidermis (figure 3M; electronic supplementary material S5) after abolition of the Turing-like patterning of reticulate scales (figure 3G–L). Given that tissue folding is affected by both the relative growth rates and the specific material properties of each constituent layer [21], we further test the mechanical folding hypothesis by experimentally modifying epidermal stiffness in developing chicken embryos. Previously, hydrocortisone treatment has been shown to trigger substantial differentiation and surface keratinization of embryonic chicken skin cultures [48]. Therefore, we treated developing chicken embryos at E13 with a single intravenous injection [34] of hydrocortisone, either in isolation or together with SAG treatment (figure 4; table 2; electronic supplementary material, figure S6).

**Table 2.** Summary of replicates of combined SAG and hydrocortisone injections (figure 4; electronic supplementary material, figure S6).

hydrocortisone concentration (µg)	SAG concentration (µg)	survival (#)	treatment effect (%)
0	0	7/8	100
50	0	6/8	100
200	0	6/9	100
10	400	5/9	100
50	400	6/9	100
100	400	5/9	100
200	400	5/8	100

When treated with 50 μg hydrocortisone, embryos develop reticulate scales that lack their normal elevation and definition, most notably on the second and fourth toepads (figure 4B). The digits of embryos treated with 200 μg hydrocortisone exhibit an even greater restriction of reticulate scale development, with the second and fourth toepads remaining broadly flat (figure 4C). H&E staining of sections of the fourth toepads reveals substantially increased epidermal keratinization associated with hydrocortisone treatment (figure 4B,C) [48]. As already shown above (figure 2), samples treated with 400 μg SAG exhibit substantial labyrinthine folding (figure 4D). When embryos are treated with a combination of a constant dose (400 μg) of SAG and a variable dose (10, 50 or 100 μg) of hydrocortisone, we observe an increasing reduction in the depth of the SAG-induced labyrinthine folds (figure 4E–G). When using yet a higher dose (200 μg) of hydrocortisone, the surface of the second and fourth toepads appear almost completely smooth (figure 4H) despite the SAG treatment. H&E staining of sections of the fourth toepad (figure 4) indicate that increased doses of hydrocortisone strongly promote keratinization and thickening of the epidermis. Note also that, remarkably, hydrocortisone-induced hyper-keratinization displaces SAG-induced elastic instabilities from the surface of the epidermis to the epidermal–dermal boundary, which therefore exhibits large undulations. Overall, these experiments demonstrate that hydrocortisone treatment triggers additional keratinization and thickening of the epidermis, thereby modifying its material properties. This modification is sufficient to (i) reduce the normal elevation of reticula in embryos that have not been treated with SAG and (ii) mechanically restrict the emergence of SAG-induced surface folds.

### 2.6. Mechanical growth simulations recapitulate folding patterns

**Figure 5.**
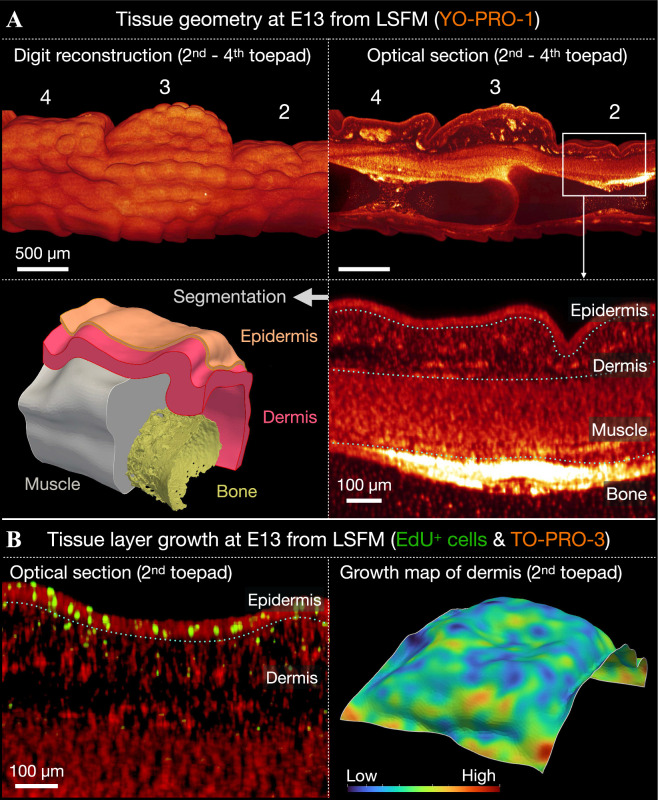
Building a mechanical growth simulation model. Using segmented volumetric LSFM, we build a numerical model to test whether restricted growth-driven mechanical instability can produce folding patterns in the absence of a Turing-like chemical self-organizational process. (A) Untreated chicken digit imaged at E13, stained with YO-PRO-1 to label cell nuclei (top-left panel). This reveals clear differences in signal intensity between the epidermis, dermis, muscle and bone across individual toepads, allowing us to segment these individual tissue layers (top-right and bottom-right panels) and build a 3D model of the toepad (bottom-left panel). (B) EdU-positive cells within the epidermis and dermis of an untreated sample at E13 (left panel) are used to estimate the relative amount and spatial distribution of growth within these tissue layers (right panel). This reveals broadly homogeneous growth within the dermis and epidermis, but a greater growth of the latter versus the former.

We next sought to further verify our experimental findings and test whether growth-driven mechanical instability alone, constrained by the parameters that we can measure on the relevant embryonic material, is sufficient to trigger the labyrinthine folding patterns observed in our SAG-treated chicken embryos (figure 2F–I). Therefore, we build a mechanical growth numerical model [18] using volumetric image data acquired from chicken embryos (figure 5). First, we acquire high-resolution data regarding the precise tissue layer geometry of individual toepads (figure 5A, top row). Chicken digits fixed at E13 were imaged using LSFM after staining with YO-PRO-1 iodide to label cell nuclei. Optical LSFM sections reveal recognizable differences in the signal intensity of the YO-PRO-1 fluorescence across different tissue layers of the individual toepads, including the epidermis, dermis, muscle and bone (figure 5A, bottom right panel). This allows us to segment these individual tissue layers and create a 3D toepad model (see §4 for further details) (figure 5A, bottom left panel). Second, after detecting EdU-positive cells with LSFM in the third digit at E13 (figure 5B, left panel), we estimate the relative amount and spatial distribution of growth within these tissue layers and build a 3D growth map (figure 5B, right panel). This reveals broadly homogeneous tissue growth within each of these two tissue layers, while the overall growth of the epidermis was 23% higher than that of the dermis.

**Figure 6.**
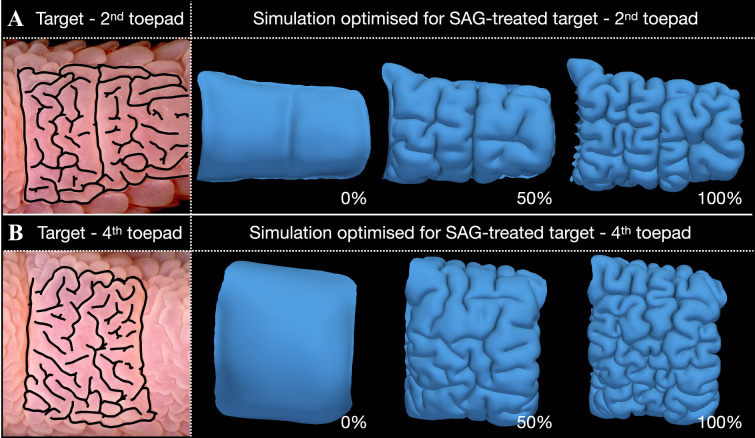
Mechanical growth simulations. (A,B) The parameters of the mechanical model are optimized using the normalized two-component metrics (see §4) of target networks of the skin surface geometry (manually segmented from 2D images; left column) acquired from the second (A) and fourth (B) toepads of SAG-treated embryos at E18. The right panels show the progression of the optimized simulations. See electronic supplementary material, videos S1–S2 for the full simulations.

Using this segmented LSFM data, we build a numerical model to simulate the mechanical growth of the skin on both the second and fourth toepads of the third digit (see §4). We then manually mark the networks of folds at the ventral toepad surface on high-resolution 2D images of SAG-treated chicken embryos at E18 (left panels of figure 6A,B). Using these networks as targets, we implement a Bayesian optimization process to estimate the best parameters for our mechanical model. In other words, the process runs iterative simulations until the simulated output provides an optimal match with the target networks (see §4 for further details). This approach yields simulation parameters that qualitatively recapitulate the labyrinthine folding patterns observed in SAG-treated embryos (right panels of figure 6A,B; electronic supplementary material, videos S1–S2).

It is remarkable that to achieve the appropriate skin surface labyrinthine folding observed on the digits of SAG-treated embryo, our Bayesian optimization process produces increased epidermal growth, stiffness and Poisson’s ratio (see table 3), all of which are expected to occur under upregulation of Shh pathway signalling triggered by SAG treatment. As discussed above, we experimentally confirmed that the epidermis of SAG-treated samples experiences a significant increase in stiffness (figure 3M) and growth (electronic supplementary material, figure S5) relative to controls. Overall, these results demonstrate that, in the complete absence of gene signalling, a mechanical system can explain the emergence of skin surface folding of the embryonic chicken toepad.

**Table 3.** Bayesian-optimized values (followed by rounding) of the effective parameters used in simulations.

	$E_{epidermis/dermis}$	$\nu_{epidermis/dermis}$	$\lambda_{T,epidermis/dermis}$
control 2nd and 4th toepad (electronic supplementary material, S7)	1/1	0.15/0.15	1.48/1.2
treated 2nd and 4th toepad (figure 6A,B)	3/1	0.35/0.15	1.55/1.2

Note that when we optimize the model parameters using skin surface pattern networks acquired from control DMSO-treated chicken embryos at E18 (i.e. with normal nodular reticula), the optimal growth model produces polygonal domains (electronic supplementary material, figure S7 and videos S3–S4). However, these domains are on average at least twice as big as real reticulate scales. Furthermore, to achieve this pattern, our Bayesian optimization process must assign equal effective epidermal and dermal stiffnesses (see table 3). Such effective parameters are biologically inaccurate, as they are not representative of the true material properties of embryonic chicken skin, which has a substantially stiffer epidermis than dermis. This discrepancy is unsurprising, as the normal patterning of reticulate scales is not dominated by a self-organized mechanical process of skin folding, but instead follows a template pattern with a length scale determined by a Turing-like chemically mediated reaction-diffusion process.

### 2.7. Swellable PDMS bilayer experiments recapitulate constrained growth-driven folding

**Figure 7.**
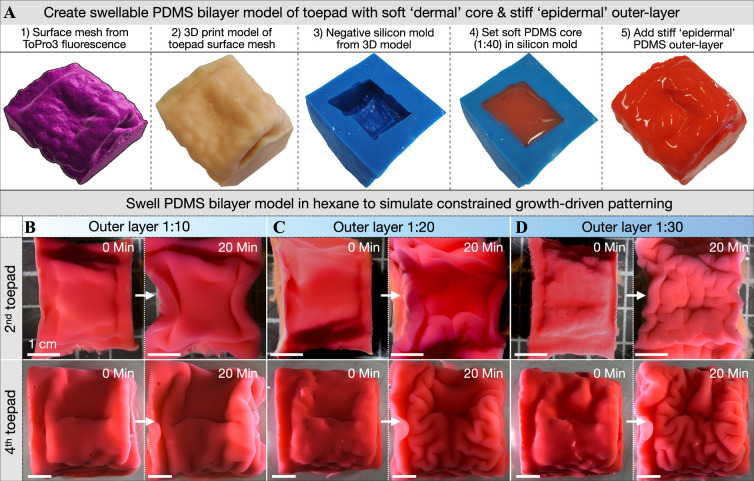
Swellable PDMS bilayer models recapitulate constrained growth-driven folding. (A) TO-PRO−3 nuclear fluorescence was captured with LSFM to create a surface mesh of the second and fourth toepads of an E13 chicken embryo. This mesh was 3D-printed and used to create a negative silicone mould, into which a lightly cross-linked PDMS ‘dermal’ core was set. This core was partially cured and drip-coated with a more strongly cross-linked ‘epidermal’ outer layer. Models were submerged in hexane for 20 min to simulate constrained growth. (B) Models with a strongly cross-linked outer layer (1 : 10) do not exhibit folding. (C,D) Models with a less strongly cross-linked outer layer (1 : 20 or 1 : 30) exhibit substantial labyrinthine folding of the surface. Therefore, constrained growth can generate folding patterns in entirely physical systems. See electronic supplementary material, videos S5–S10 for full experimental results.

To further validate our results, we undertook physical experiments to induce constrained growth using PDMS gel bilayer models. First, using nuclear-staining data from LSFM, we created surface meshes of both the second and fourth toepads of chicken embryos at E13 (figure 7A, left panel). These meshes were 3D printed and used to create negative silicon moulds, into which a lightly cross-linked PDMS ‘dermal’ core was set (figure 7A, second, third and fourth panels). This soft core was then drip-coated with a more strongly cross-linked ‘epidermal’ outer layer of PDMS (figure 7, rightmost panel; see §4 for further details). When submerged in hexane, PDMS gel models swell. As the hexane penetrates from the surface, there is initially greater swelling at the outer layer relative to the interior, which results in growth-driven compression and folding, thereby mimicking our experimental (figures 2 and 4) and computational (figure 6) results. This technique has previously been used to recapitulate the development of cortical convolutions of the brain [22].

Next, we test the effect of variation of material properties on mechanical folding. The soft core was consistently cross-linked at a concentration ratio (i.e. volume cross-linker to volume PDMS ratio) of 1 : 40, whereas the cross-linking of the outer layer was altered to examine the effects of its stiffness upon pattern emergence. Bilayer models with the most strongly cross-linked outer layer (1 : 10) do not exhibit notable folding, although we observe a slight increase in the overall size of the model and some deformation of the surface (figure 7B electronic supplementary material, videos S5–S6). On the other hand, bilayer models with a less strongly cross-linked swelling outer layer (1 : 20 or 1 : 30) exhibit labyrinthine surface folding (figure 7C,D; electronic supplementary material, videos S7–S10). The folding observed in these bilayer models is qualitatively comparable with the folding induced by SAG (figure 2) and combined SAG/hydrocortisone treatments (figure 4), as well as to our numerical simulations (figure 6). Note that the PDMS toepad models exhibit an artifactual central depression due to dehydration of the samples imaged with light-sheet microscopy. Overall, these results demonstrate that, in entirely physical models of the chicken embryo footpads, comparable labyrinthine folding patterns with those observed in SAG-treated samples can arise solely from constrained growth.

## 3. Discussion

Our results demonstrate that transient exacerbation of sonic hedgehog pathway signalling in chicken embryos at E12 or E13 abolishes the Turing-like patterning of ventral footpad reticulate scales (figure 3) [35,42] and promotes overall epidermal growth and stiffness. This causes a transition to mechanical labyrinthine folding of the skin surface (figures 2 and 4). Both our computer simulations (figure 6) and PDMS bilayer experiments (figure 7) confirm that the labyrinthine patterns observed on the toepads of SAG-treated embryos can be achieved in entirely mechanical systems. Similarly to the morphogenesis of other tissues in diverse contexts, such as brain gyrification [22], the emergence of crocodile head scales [15,18] or of polygonal domains on the naked nose of multiple mammalian species [17], and the development of chicken intestinal villi [23,24], our results show that constrained growth can play a substantial role in the morphogenesis of the skin and its appendages [15]. Remarkably, the same SAG treatment at E11 (instead of E12 or E13) does not abolish the Turing-like patterning of ventral footpad skin appendages but triggers a permanent reticulate-scale-to-feather transition, highlighting the very dynamical nature and stage-sensitivity of the gene regulatory network during development.

It has been proposed that ‘generic forces’, meaning those applicable to both living and non-living systems, such as tissue mechanics, may have played an important role in defining the morphologies of early multicellular life, with gene regulatory networks (GRNs) later stabilizing and refining such morphological characters [49,50]. In this respect, it is conceivable that the patterning of reticulate scales first arose from mechanical instability, determined by the differential growth and material properties of the skin, and were subsequently stabilized and further refined by a chemical system of diffusing and interacting morphogens, including *Shh* (figure 1D). This scenario, although very speculative, is compatible with the observations that (i) the normal development of a reticulate scale does not clearly involve the presence of an anatomical placode, but is clearly associated with a ‘signalling placode’ [35,42] (involving the Shh and β-catenin pathways; figures 1D and 3A–B) and (ii) patterning of (irregular) polygonal reticulate scales is attainable via a purely mechanical system of constrained growth (electronic supplementary material, figure S7).

Importantly, the emergence of folding is restricted in our PDMS bilayer models with stiffer outer ‘epidermal’ layers (figure 7B,E). This bears notable similarity to our combined SAG and hydrocortisone treatments, in which the deposition of additional keratin is sufficient to restrict the folding of the skin surface (figure 4). Hence, our experiments indicate that natural variation of material properties provide a simple means for producing a broad diversity of folding patterns in developing embryos. In addition to Turing-like chemical patterning, such mechanical systems have undoubtedly contributed to the evolution of diverse morphogenetic processes.

## 4. Methods

### 4.1. Animal husbandry

Fertilized broiler chicken eggs (La Prairie 9, 1721 Cournillens, Switzerland) were incubated at 37.5°C with approximately 40% relative humidity. Maintenance of, and experiments with, all chicken embryos were approved by the Geneva Canton ethical regulation authority (authorization GE244) and performed according to Swiss law.

### 4.2. *In situ* hybridization

Dissected digits from chicken embryos were fixed overnight in 4% paraformaldehyde (PFA) at 4°C prior to graded dehydration into methanol (MtOH). Whole-mount *in situ* hybridization (WMISH) was undertaken as previously described, following a bleaching stage to remove pigments [51]. Following WMISH, samples were post-fixed in 4% PFA and imaged using a Keyence VHX 7000 digital microscope. Next, samples were embedded in optimal cutting temperature (OCT) compound and sectioned at 15 µm with a cryostat (Leica CM1850). Sections were imaged with an automated slide scanner (3DHISTECH).

### 4.3. Haematoxylin and eosin staining

Fixed samples were dissected, embedded in paraffin and processed for H&E staining as previously described [51]. Paraffin sections were acquired at 10 µm with a microtome (Leica RM2255). Stained samples were mounted using UltraKit mounting medium and imaged with an automated slide scanner (3DHISTECH).

### 4.4. *In vivo* intravenous drug treatments

Intravenous injection was undertaken as previously described [34,35]. Eggs at E12 and E13 were cleaned with 70% ethanol (EtOH). Next, the eggs were candled to identify suitable veins for injection. A detailing saw (Micromot 50/E, Proxxon) was used to cut a small window in the eggshell around the selected vein, while keeping the underlying egg membrane intact. The eggshell was then removed with fine forceps, and mineral oil was applied to the exposed membrane, enabling clear observation of the vein under a dissecting microscope. A Hamilton syringe attached to a micromanipulator (MM33 right, Marzhauser) was used to deliver 30 µl of a solution containing 300 or 400 µg of SAG (Selleckchem) at E12 or E13, respectively, dissolved in dimethyl sulfoxide (DMSO). This equates to an equivalent dose of about 40 µg SAG per gram of embryo weight at both stages. Patent blue was also added to the solution, enabling visualization during injection. Control embryos were injected with 30 µl DMSO and patent blue. Treatment of embryos with hydrocortisone (Selleckchem) and combinations of SAG and hydrocortisone in different concentrations, delivered in a single injection, was performed following the same intravenous injection method. After injection, clear adhesive tape was applied to the window to prevent infection, and the eggs were returned to the incubator. Prior to fixation, embryos were treated with an injection of 5-ethynyl-2′-deoxyuridine (EdU) to label proliferating cells (baseclick). Embryos were subsequently fixed overnight in 4% PFA at the desired stage. See the tables below for details regarding the number of replicates (see electronic supplementary material, figures S1 and S6) of the experiments shown in figures 2 and 4.

### 4.5. Nanoindentation

Nanoindentation was used to acquire stiffness measurements (effective Young’s modulus) of embryonic chicken toepad skin at different stages of treatment, using a Piuma nanoindenter (Optics11). Embryos were injected at E13 with either 400 µg SAG or DMSO as a control, and dissected at either E13 plus 5 h, E15 or E17. Embryos were indented prior to fixation. A total of seven embryos were used for each treatment at each time point. Each foot was positioned with the ventral side upwards, and the fourth toepad of the middle digit was indented at six points in a 3 × 2 grid separated by 120 µm with each movement in the *x* or *y* direction. Samples were indented with a tip of 108.5 µm radius and 0.54 N m^−1^ stiffness at a depth of 1.5 µm. Only measurements from load-displacement curves with a Hertzian contact model fit of ≥95% were analysed. Data were log transformed to normalize the distribution prior to statistical analysis. Mean effective Young’s m odulus values for individual samples, as well as the overall mean across mean values of different samples (and standard deviation) are presented in figure 3M. An ANOVA including both embryo stage (E13 plus 5 h, E15 or E17) and treatment (SAG or DMSO) as fixed effects was implemented. A Tukey post hoc test was used to compare all group means. Statistical analysis was undertaken in R [52].

### 4.6. Light sheet fluorescence microscopy

Samples in MtOH were rehydrated and permeabilized for 24 h in phosphate-buffered saline with gelatine, sodium azide, saponin and TritonX100 (PBSGST). Samples were then incubated in TO-PRO-3 Iodide to stain cell nuclei (2:1000, ThermoFisher). EdU-positive cells were detected after labelling according to manufacturer guidelines (baseclick). Following staining, samples were dehydrated into MtOH, cleared in accordance with the iDISCO protocol [53], and imaged with a light sheet microscope (Ultramicroscope Blaze, Miltenyi Biotec). The second and fourth toepads were imaged at high resolution with a 12× objective lens.

### 4.7. Segmentation of LSFM data

LSFM data segmentation was undertaken in accordance with our previous study regarding crocodile head scale patterning via compressive folding of the skin [18]. The epidermis, dermis and muscle layers were segmented using the YO-PRO-1 signal, whereas the bone was segmented using Alizarin Red staining. The segmented data was used to build a finite element model (FEM, see below) of the second and fourth toepads. Cell nuclei staining signal allows precise segmentation of the epidermis from the dermis because the former exhibits a higher cell density. More specifically, the 3D image generated by LSFM on the basis of the YO-PRO-1 fluorescence signal was subjected to 3D Canny’s edge detection [54] in MATLAB R2021a, generating a 3D binary image in which nonzero voxels form point clouds corresponding to two 3D surfaces: the surface of the epidermis and the epidermis–dermis boundary. For each of these two surfaces, we compute at each point the surface normal vector from the intensity gradient. The position of points and their corresponding normal vectors are then fed to a screened Poisson surface reconstruction algorithm [55] in Meshlab [56] to reconstruct triangular surface meshes, which effectively represent the initial point clouds in a much lighter format: 3D meshes are much easier to manipulate (e.g. with the Laplacian smoothing algorithm to filter out the artifactual stair-step patterns in the original voxelized data format). The epidermis surface and the epidermis–dermis boundary allow computing the epidermis thickness across each control and treated sample at different developmental stages.

For segmenting muscle tissue, we used a semi-automatic procedure based on the YO-PRO-1 fluorescence signal. We (i) choose about 20 sections in each of the three (*x*, *y* and *z*) directions and manually mark the separation between the dermis and the muscle tissue, (ii) store the coordinates of all profile points as a 3D point cloud and compute their normal with variational implicit point set s*urface* [57], and (iii) use screened Poisson surface reconstruction [55] from Meshlab [56] to generate a mesh corresponding to the dermis–muscle interface. Then, epidermis, dermis and muscle surface meshes are smoothed using the Blender software (https://www.blender.org) to remove artefacts caused by dehydration. Finally, volumetric tetrahedral meshes are generated from smoothed surfaces using the program TetGen (http://www.tetgen.org).

EdU-positive (EdU+) cells are segmented after computing the principal curvatures of the 3D signal [58]. This approach is highly efficient for individually segmenting cells when they are grouped (i.e. in contact). As the signal intensity is embedded in a 3D domain, three signal principal curvatures $k_{1,2,3}$ are computed (in MATLAB R2021a) for each voxel, and voxels characterized by $k_{s}>k_{threshold}$, where $k_{s}={\left(k_{1}^{+}k_{2}^{+}k_{3}^{+}\right)}^{1/3}$ and $k_{i}^{+}=max(k_{i},0)$, are stored. The centroid of the connected voxels is considered as the location of an EdU+ cell. We then compute the density of EdU+ cells, separately for the dermis and the epidermis, by choosing sampling points in the corresponding segmented tissue layers. The space surrounding each sampling point is limited to a box of 80 × 80 × 80 voxels clipped by the layer boundaries. The density of EdU+ cells at a sampling point is computed as the number of cells inside the clipped box divided by its volume. Our analysis on the EdU+ cell density distribution in an untreated sample at E13 (figure 5B) evidences a broadly homogeneous growth within dermis and epidermis, but larger overall cell proliferation (by 23%) in the latter.

### 4.8. Differential growth and neo-Hookean material model

In short, we define the bulk of the material as the collection of points forming a specific configuration in 3D space. The motion of these points fully describes its deformation. We define the deformation gradient, F, as a second-order tensor which relates the initial (reference) configuration of any material point X to its current (deformed) configuration x :


(4.1) $$F=\frac{dx}{dX}$$

To model tissue growth, the deformation gradient tensor F is decomposed into elastic and growth components $F_{e}$ and $F_{g}$, respectively :


(4.2) $$F=F_{e}F_{\mathrm{g,}}$$

Here, $F_{g}$ describes the effective growth of the tissue and can be derived from the biological experiments/observations. In our model, we assume that the growth deformation gradient is saturating as a function of time :


(4.3) $$F_{g}\left(t\right)=I+\lambda_{N}\left(1-exp\left(-\beta t\right)\right)N⊗N+\lambda_{T}\left(1-exp\left(-\beta t\right)\right)\left(I-N⊗N\right)$$

in which, N is the normal vector in the reference configuration, β>0 is a constant that determines growth rate and I is the identity matrix. Furthermore, $\lambda_{N}$ and $\lambda_{T}$, determine the final relative growth in the normal and tangential directions, respectively.

Neo-Hookean material models (a sub-group of *hyper-elastic* models) are appropriate for soft material (bio-)mechanics because they are robust even under large deformations [59]. In hyper-elastic models, the stress tensor is computed from the *strain energy density function*:


(4.4) $$\Psi =\frac{\mu }{2}\left(tr\left(F_{e}F_{e}^{T}\right)J^{-\frac{2}{3}}-3\right)+K\left(J-lnJ-1\right),$$

where $J=detF_{e}$ and μ and K are shear and bulk moduli, respectively, which are related to Young’s modulus E and Poisson’s ratio ν as follows:


(4.5) $$\mu =\frac{E}{2\left(1+\nu \right)}\;\;\text{and}\;\;K=\frac{E}{3\left(1-2\nu \right)}.$$

In the framework of hyper-elastic materials, we compute the Cauchy stress σ directly from the strain energy density function:


(4.6) $$\sigma =\frac{1}{J}\frac{\partial \Psi }{\partial F_{e}}F_{e}^{T}$$

Substituting (4.4) into (4.6) results in the following stress function


(4.7) $$\sigma =\mu J^{-5/3}dev\left(F_{e}F_{e}^{T}\right)+K\left(1-\frac{1}{J}\right)I.$$

Much more detailed information regarding this mechanical growth model is provided in our previous study investigating crocodile head scale development [18].

### 4.9. Numerical simulations and parameter optimization

In order to perform numerical simulations, the mechanical model formulation described above is discretized for tetrahedral elements using the FEM and integrated with contact and viscous forces. The steady-state solution of the displacement field is then computed by solving Newton’s second law of motion. The model is implemented in an in-house application which exploits NVIDIA GPUs for high performance computation. For that purpose, we used the CUDA programming language to develop intensive-computation kernels, whereas C++ is used for data management, geometry processing and input/output operations. Our application integrates the following open-source libraries: CUDA C++ Core Libraries (https://github.com/NVIDIA/cccl, Apache-2.0, FreeBSD, BSD-3-Clause licences) for parallel algorithms, Eigen (https://gitlab.com/libeigen/eigen, MPL-2.0, BSD licences) for linear algebra, and libigl (https://github.com/libigl/libigl, GPL-3.0, MPL-2.0 licences) for geometry processing. The simulation input is a tetrahedral mesh which defines the geometry of the chicken toepads (epidermis, dermis and muscle layers). Also, a set of model parameters are used, including the Young’s modulus, Poisson’s ratio and the normal/tangent growth values for each layer. The deformation of the skin is then computed and the final geometry is generated as a tetrahedral mesh.

In order to optimize the parameter values associated with each skin layer, the mechanical model is integrated with a Bayesian optimization process (bayesopt library from MATLAB R2021a with parallel sampling; i.e*.* a machine-learning global minimization algorithm). In short, the optimality criterion consists of the distance between the 2D projection of the folding network of the simulated surface meshes *versus* the manually segmented skin surface pattern imaged on embryonic toepads with a Keyence VHX 7000 digital microscope. Each folding network is characterized by two-component metrics consisting of the sum of the domain (cycle) perimeters and the sum of incomplete edge lengths normalized by the length of the toepad. Given that components within metrics vectors may differ significantly in absolute values, we need to normalize them properly. For this purpose, we first extract the metrics of four samples (second and fourth toepads of a control and a treated sample at E18) and compute, for each component, the corresponding inter-sample mean and standard deviation. Components of all analysed samples are normalized by subtracting the inter-sample mean and dividing by the inter-sample standard deviation. To extract the folding networks from the simulated surface geometries, we first compute the minimum principal curvature of the epidermis surface mesh and then segment the skin folds by applying a skeletonization algorithm [60] on the low curvature regions of the mesh, followed by graph simplification (using MATLAB R2021a).

Finding optimal mechanical parameter values for control and treated targets is performed in two steps. First, we use the fourth toepad control sample to perform optimization on the five-dimensional parameter space including epidermis Young’s modulus ($E_{epidermis}$; keeping $E_{dermis}=1$), epidermis and dermis Poisson’s ratios ($\nu_{epidermis/dermis}$) and tangential growth values ($\lambda_{T,epidermis/dermis}$). Second, using a SAG-treated fourth toepad sample, we perform another optimization on the three-dimensional parameter space including epidermis-related parameters (i.e. $E_{epidermis}$, $\nu_{epidermis}$ and $\lambda_{T,epidermis}$) while keeping the two other parameters unchanged. Given that muscle is a much stiffer tissue than dermis and epidermis, we assume that it behaves as a rigid material. The optimization process, which typically takes a few thousand iterations, is performed until no more improvement is observed in the last 500 iterations. See table 3 for the complete list of parameter values. More detailed information regarding numerical simulations and parameter optimization are provided in our previous study investigating crocodile head scale development [18].

### 4.10. Simulations of constrained growth with PDMS bilayer models

Swellable polydimethylsiloxane (PDMS) bilayers were used to simulate epidermal constrained growth [22]. First, surface meshes of the second and fourth E13 toepads were created from TO-PRO-3 nuclear fluorescence acquired from light sheet microscopy (Ultramicroscope Blaze, Miltenyi Biotec). These meshes were 3D printed and used to create negative silicone moulds (Mold Star 30 kit, Smooth-On). PDMS models of the dermis (crosslinker added at a ratio of 1 : 40) were set in these moulds. The PDMS solutions were degassed to remove bubbles, and the soft core was set in the moulds for approximately 90 min at 75°C. After partial curing, the stiff outer layer was added by drip-coating (cross-linker was added at a ratio of 1 : 10, 1 : 20 or 1 : 30), followed by partial curing for 15 min. Three drip-coated layers were applied to each model to achieve adequate thickness. The entire model was then cured overnight at 65°C. To simulate constrained growth, the PDMS bilayer model was submerged in hexane for 20 min and imaged every 2 s using an overhead-mounted Nikon D850 camera. The full results are shown in electronic supplementary material, videos S5–S10.

## Data Availability

All data required to evaluate the conclusions in this paper are present in the paper and/or the supplementary materials [61]. Additional data related to this paper may be requested from the authors. The executables and original code used for the numerical simulations presented here are provided as a Code Ocean capsule at https://codeocean.com/capsule/4902320/tree/v1.
